# Dendritic cells transduced with glioma-expressed antigen 2 recombinant adenovirus induces specific cytotoxic lymphocyte response and anti-tumor effect in mice

**DOI:** 10.1186/s12950-020-0239-6

**Published:** 2020-01-31

**Authors:** Gaohai Shao, Changlong Zhou, Kunlong Ma, Wang Zhao, Guibo Feng, Qijiang Xiong, Ling Yang, Zhao Yang

**Affiliations:** 1 Department of orthopedics, Yongchuan Hospital, Chongqing Medical University, Chongqing, 402160 China; 2 Department of Neurology, Yongchuan Hospital, Chongqing Medical University, Chongqing, 402160 China

**Keywords:** Dendritic cells, GLEA2, CTL, Anti-tumor

## Abstract

**Introduction:**

Glioma is an aggressive common cancer with high mortality worldwide. Up to date, the effective medical therapeutical strategy is limited. Numerous previous studies have indicated that glioma-expressed antigen 2 (GLEA2) might be an attractive prognostic glioma biomarker.

**Methods:**

In this experiment, dendritic cells (DCs) transduced with GLEA2 recombinant adenovirus were utilized to generate cytotoxic lymphocytes (CTLs) in vitro. Additionally, trimera mice were immunized with the transduced DCs to generate CTLs in vivo.

**Results:**

The data demonstrated that GLEA2 transduced DCs could effectively generate specific CTL response against glioma without lysing autologous lymphocytes. Moreover, GLEA2 transduced DCs significantly attenuated the tumor growth and prolonged the life span of tumor bearing mice.

**Conclusions:**

These findings suggested that DCs transduced with GLEA2 recombinant adenovirus could generate effective CTL mediated anti-tumor response, and might represent insight in glioma therapy.

## Introduction

Gliomas are the most common primary central nervous system tumors [1–3]. Glioblastomas represent 50% of all gliomas in adults with an extremely poor prognosis [4–6]. Despite innovative therapeutic methods such as surgical resection, chemotherapy, and radiation therapy, the survival ratio is very low [7–9]. Therefore, the novel therapeutic approaches are in great need.

GLEA2 was identified as a novel antigen termed glioma-expressed antigen 2 (GLEA2) inducing immune response in glioma patients [10]. The evidence found an immune response against GLEA2 in 17 patients (43%). In addition, screening with allogenic sera from other glioma patients revealed GLEA2 directed antibodies in two out of five pilocytic astrocytomas and in one out of two astrocytomas [11]. In serological analyses by the SEREX (Serological analysis of expression cDNA libraries) technology, GLEA2 were found to frequently elicit immune responses in sera of GBM patients and acted as a prognostic biomarker for glioma [12]. Therefore, GLEA2 may be act as a new target in cancer therapy.

Specific CTLs mediated cellular immunotherapy has been utilized to treat malignant tumors [13–15]. Antigen presentation is an important step to elicit adaptive immune response. Dendritic cells are the potent professional antigen-presenting cells and have the robust antigen-presenting efficiency [16–18]. They can present a tumor associated antigen to the immune system and generate specific immune response. Dendritic cells transduced with various tumor associated antigens (TAAs) can initiate specific antitumor effects in vitro and in vivo [19–21].

However, the related evidence linking GLEA2 to glioma therapy has not been reported. In this experiment, dendritic cells transduced with GLEA2 were constructed and the capability of inducing immuno- responses was detected. We found that GLEA2 transduced DCs could elicit specific CTL response against glioma without lysing autologous lymphocytes. In addition, GLEA2 transduced DCs could also inhibit the tumor growth and improve the life span of tumor bearing mice.

## Materials and methods

### Mice, cells, and other reagents

NOD/SCID and BALB/c mice were purchased from the Laboratory Animal Institute of Beijing Medical University and were used at 6 weeks of age. Animals were bred in the Laboratory Animal Center and all studies were performed in agreement with the local ethics committee. The glioma cell line U251, was purchased from American Type Culture Collection. The cell line was maintained as monolayers in Dulbecco’s modified Eagle’ medium containing 10% heat- inactivated fetal bovine serum kept at 37 °C in a humidified atmosphere of 5% CO_2_ in air.

### Construction of recombinant adenovirus encoding GLEA2

The recombinant adenovirus vector encoding murine GLEA2 was constructed using the Adeno-XTM Expression System (Clontech, Palo Alto, CA, USA) according to the manufacturer’s instructions. Briefly, the GLEA2 cDNA was cloned into the shuttle vector pDC315 and sequenced. The desired replication-deficient adenovirus containing the full length cDNA of GLEA2 was generated by homologous recombination through co-transfection of plasmids pDC315-GLEA2 and pBHG1oXE1, 3Cre in HEK 293 cells using the DOTAP liposome reagent (Roch, Germany). After several rounds of plaque purification, the adenovirus containing the GLEA2 gene was amplified and purified from cell lysates by banding twice in CsCl density gradients. Viral products were desalted and stored at -80 °C in phosphate buffered saline (PBS) containing 10% glycerol (v/v). The infectious titer was determined by a standard plaque assay. A second recombinant, El-, E3-deleted adenovirus carrying the LacZ protein under the control of CMV promoter (Ad-LacZ), was used as a control vector.

### Preparation of Trimera mice

Male nonobese diabetes/severe combined immunodeficiency (NOD/SCID) and BALB/c (H-2d) mice, 6–10 weeks old at the onset of experiments, were purchased from the Institute of Animal of Beijing Medical University (Beijing, China). During the course of experiments, the mice were kept in pathogen free animal facilities with controlled temperature and humidity, under a 12-h light/dark cycle, and with food and water containing cyprofloxacin (20 l g/ml). All animals were acclimated for at least 1 week before the experiments. Animal care and use were performed in accordance with the guidelines of the Dutch Committee of Animal Experiments. Recipient BALB/c mice received a lethal dose of total body irradiation (i.e., day 0, 3.5 Gy and day 3, 9.5 Gy). On days 4–6, 3 × 10^6^ mixed bone marrow cells (in 0.2 ml PBS) from NOD/SCID mice were transferred into each irradiated recipient by i.v. injection. One day after bone marrow infusion, each recipient mouse was.

injected (i.p.) with 2× 10^8^ human PBMCs (HLA-A2). All mice were kept under specific pathogen-free conditions, fed with sterile food and acid water containing cyprofloxacin (20 μ g/ml).

### Generation of DCs

PBMCs were obtained by Ficoll-Hypaque (Celbio S.P.A., Italy) gradient separation of buffy coats of heparinized blood samples collected from HLA-A(*)02.01-typed healthy human donors who provided written informed consent. The DCs used for in vitro CTL stimulation were generated from autologous PBMCs (10^7^ cell/mL) seeded in complete RPMI-1640 medium with the addition of 5% heat-inactivated human AB serum, 2 mM L-glutamine and 100 U/mL penicillin/streptomycin at 37 °C in 5% CO_2_ humidified atmosphere for 4 h. Nonadherent cells were removed, whereas adherent cells where cultured for 7 days in medium containing 50 ng/mL of granulocyte-macrophage colony stimulating factor (GM-CSF) and 0.05 ng/mL of interleukin-4 (IL-4) (both purchased from R&D System, Minneapolis, MN); in all cases, the medium containing GM-CSF and IL-4 was replaced every 48 h. FACS analysis demonstrated that DCs expressed high level of CD11c and MHC Class II (as shown in Additional file 1: Figure S1).

### Adenovirus-mediated gene transfer

Transduction of DCs with Ad-GLEA2 was done in 6-well plates with 1 × 10^6^ DCs cells/well in 3 mL RPMI-1640 medium containing 10% FBS. Virus was added to the wells at an MOI of 200 and the DCs were harvested after 24 h of incubation.

### Western blot analysis

Total cellular proteins were extracted from cultured cells using RIPA lysis buffer (50 mM Tris pH 7.5, 150 mM NaCl, 1% Nonidet P-40, 0.1% SDS, 1% sodium deoxycholate) supplemented with Protease Inhibitor Cocktail (Roche). Lysates were cleared by centrifugation at 16,000 g at 4 °C for 20 mins and supernatants containing proteins were collected. For immunoblotting, 30 μg of extracted proteins diluted in SDS-sampling buffer was resolved by SDS-PAGE (10–15%) gels and then electroblotted onto nitrocellulose membranes (Hybond ECL, Amersham Life Science). Following transfer, membranes were blocked with 5% (w/v) skim milk in TBS-Tween (TBS; 0.05 M Tris, 0.15 M NaCl, pH 7.5 and 0.1% Tween-20) for 1 h and then probed with primary antibodies diluted in 1% (w/v) skim milk powder in TBS-Tween at 4 °C overnight. Membranes were washed and then incubated with HRP-conjugated secondary antibodies and antibody reactivity was detected by the Western Lightning Ultra Detection Kit (PerkinElmer, Waltham, MA, USA) using the FujiFilm LAS-3000 Gel Documentation System (FujiFilm, Tokyo, Japan) and its associated software.

### Induction of GLEA2 specific CTLs in vitro

GLEA2-specific cytotoxic T lymphocytes (CTLs) were generated in vitro by weekly stimulation of nonadherent peripheral blood lymphocytes with irradiated autologous DCs, which were transduced with Ad-GLEA2. In brief, DCs were transduced with Ad-GLEA2 at an MOI of 200 and cultured for 2 days in fresh cytokine-supplemented medium containing 1000 U/ml of TNF-γ. They were then irradiated with 40 Gy which entirely prevented outgrowth in the control cultures. The cells were then seeded into 24-well plates at 5 × 10^4^ cells per well. The splenocytes were added at 1 × 10^6^ per well. After 7 days of co-culture with stimulators, the lymphocytes were harvested and resuspended at 5 × 10^5^ per well. The cells were then re-stimulated with 1 × 10^5^ cells per well of irradiated GLEA2 transduced DC. After 3 days, the cells were fed with 50 U/ml of IL-2. The cells were harvested 4 days later and then re-stimulated. On the 21st day, after harvesting the cells, their specificities were evaluated by a 4-h ^51^Cr release assay. Effector cells that were generated from Ad-LacZ transduced DCs or T lymphocytes stimulated by only IL-2 were used as a control.

### Chromium release assays

The presence of specific CTLs was measured in a standard 4-h chromium release assay. Briefly, target cells are labeled with ^51^Cr (100 μ Ci per 1 × 10^6^ cells; Amersham Biosciences Corp). After extensive washing, target cells are incubated with T cells at E: T ratios ranging from 100∶1 to 25∶1. All conditions were done in triplicate. Plates were incubated for 4–5 h at 37 °C in 5% CO_2_. Supernatant fluids were harvested and radioactivity was measured in a gamma counter. Percentage specific lysis was determined from the following formula: 100 × (experimental release-spontaneous release)/(maximum release-spontaneous release). Maximum release was determined by lysis of radiolabeled targets in 1% SDS.

### ELISPOT analysis

A cytokine enzyme-linked immunospot (ELISPOT) assay designed to enumerate interferon (IFN)-α-secreting cells in preparations of PBMCs was used to measure the frequency of activated T cells with the ability to detect cytokine release in response to specific antigen peptides. According to the procedure, 1 × 10^6^ splenocytes were incubated and stimulated in the wells of ELISPOT plates precoated with a high-affinity mAb to IFN-α. Subsequently, cells were washed away. The areas in which the cytokines had been bound were detected by a combination of biotinylated anti-cytokine detection mAbs and ϕ-labeled goat anti-biotin mAb. A final reagent was added to the assay in order to promote precipitation of silver on ϕ revealing the site of cytokine secretion (i.e.*,* spot formation). Fresh medium containing phytohemagglutinin (PHA, 10 μg/mL) were used as positive controls, whereas unloaded DCs in fresh medium was used as a negative control. The spots were finally evaluated by using an ELISPOT reader (A.EL.VIS GMBH, Hannover, Germany). Results were expressed as number of spots/field.

### Vaccination and tumor challenge experiments

All animal protocols were approved under guidelines of the animal protection act. Trimera mice were challenged with subcutaneously.

(s.c.) injection of 1 × 10^6^ U251 cells into the left flank to induce primary tumor model. After 10 days, Trimera mice were immunized s.c. in the base of the tail with 1 × 10^6^ transduced DCs in 100 μl PBS for three times once a week. Control mice received the same volume of PBS. The tumor volume and mean lifespan of mice were observed. Tumor volume was measured in two dimensions and calculated as follows: length/2 × width^2^.

### Adoptive transfer assay

Trimera mice were challenged with subcutaneously (s.c.) injection of 1 × 10^6^ U251 cells into the left flank to induce primary tumor model.

After 10 days, Trimera mice were injected i.v. of 1× 10^7^ lymphocytes.

Control mice received the same volume of PBS. The tumor volume and mean lifespan of mice were observed. Tumor volume was measured in two dimensions and calculated as follows: length/2 × width^2^.

### Statistics

All the experiments were run in triplicate, and the results are given as means ± SD of triplicate determinations. The statistical significance of differential findings between experimental groups and controls were determined by ANOVA and post-hoc analysis, and considered significant if *P* < 0.05. All statistical analyses were carried out with SPSS 11.5 software.

## Results

Gene induction and GLEA2 protein analysis.

**Fig. 1:**
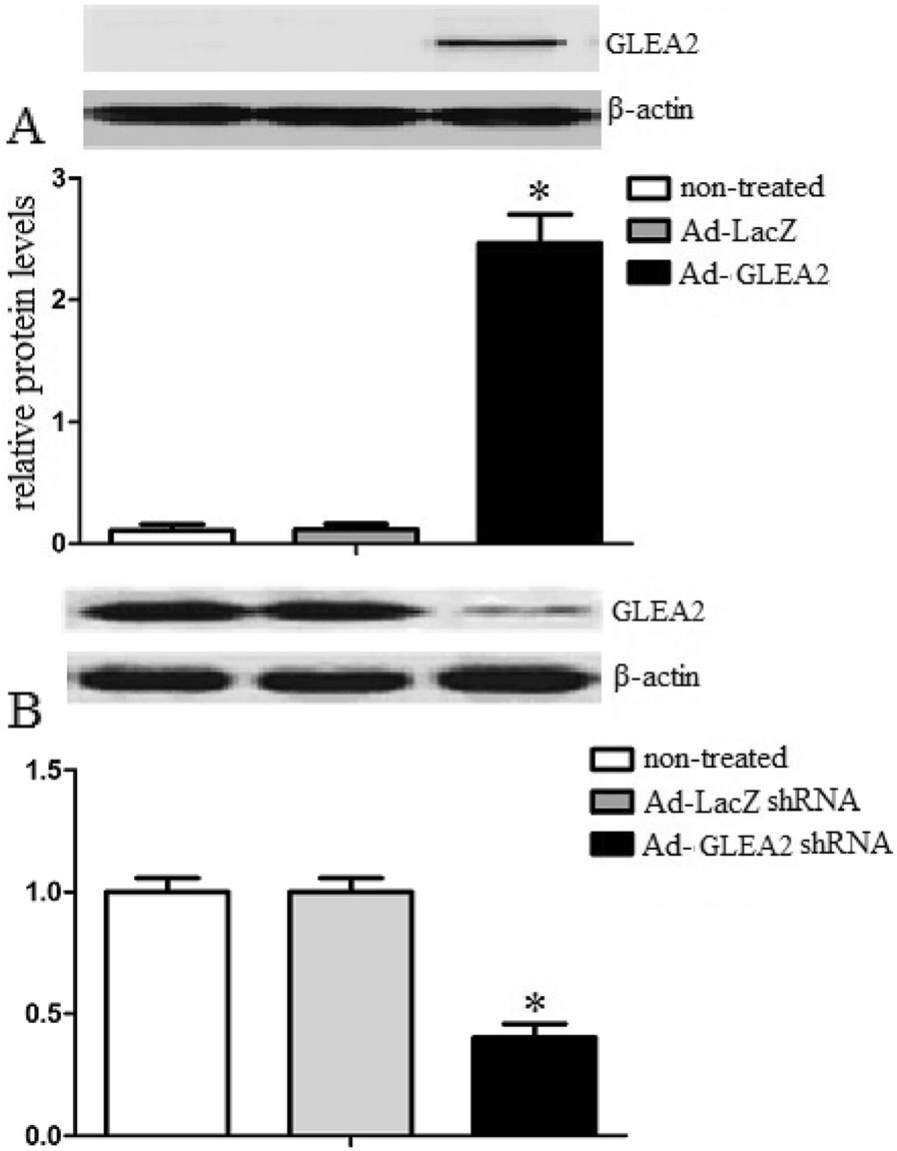
**a** Western blot assay of GLEA2 protein expression in DCs. DCs were transduced with Ad-GLEA2 or Ad-LacZ at an MOI of 200 for 24 h. The GLEA2 protein levels were analyzed by western blot assay. The GLEA2 protein could be detected after Ad-GLEA2 transduction. However, there was no expression of GLEA2 protein after Ad-LacZ transduction or in non-treated DCs. Lane 1, non-treated DCs; lane 2, DCs transduced with Ad-LacZ and lane 3, DCs transduced with Ad-GLEA2 **b** Western blot assay of GLEA2 protein expression in U251 cells. Lane 1, non-treated U251; lane 2, U251 transduced with Ad-LacZ shRNA and lane 3, U251 transduced with Ad-GLEA2 shRNA.

To detect the capability of adenovirus transduction, we analyzed GLEA2 expression of DCs by Western blot assay. DCs were transduced with Ad- GLEA2 or Ad-LacZ at MOI 200 for 24 h with protocols mentioned above. The data demonstrated that GLEA2 protein was detected after Ad- GLEA2 transduction. However, GLEA2 protein can not been detected in Ad-LacZ and non-treated DCs groups (Fig. 1a). The results suggested that Ad-GLEA2 could transduce into DCs and mediate GLEA2 protein expression. In addition, we also analyzed GLEA2 expression of U251 cells by Western blot assay. The results suggested that GLEA2 was highly expressed in U251 cells. However, Ad-GLEA2 shRNA significantly inhibited GLEA2 in U251 cells (Fig. 1b).

### Induction of GLEA2-specific CTL activity in vitro

**Fig. 2:**
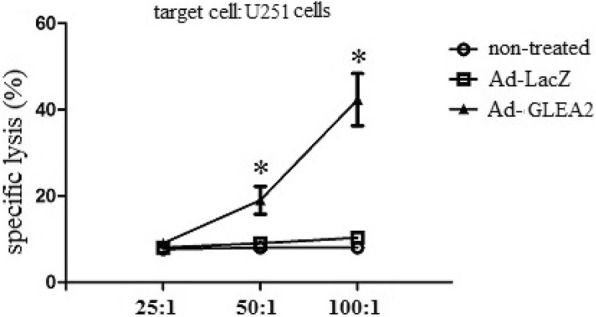
Specific lysis of target cells in vitro. GLEA2-specific cytotoxic T lymphocytes (CTLs) were elicited in vitro by weekly stimulation of peripheral blood lymphocytes with irradiated autologous DCs transduced with Ad-GLEA2. GLEA2-specific CTLs were tested against U251 cells. CTLs generated from Ad-LacZ transduced DCs and CTLs generated from non-treated DCs were used as controls. Triplicate experiments showed consistent results. Compared with controls,*P < 0.05.

To detect the capability of adenovirus transduced DCs, we analyzed GLEA2-specific CTL activity in vitro. GLEA2-specific cytotoxic T lymphocytes (CTLs) were elicited in vitro by weekly stimulation of peripheral blood lymphocytes with irradiated autologous DCs transduced with Ad-GLEA2. GLEA2-specific CTLs were tested against U251 cells or autologous lymphocytes. CTLs generated from Ad-LacZ transduced DCs and CTLs generated from non-treated DCs were used as controls. The data demonstrated that GLEA2-specific CTLs induced by Ad-GLEA2 caused greater than 40% lysis of U251 cells at an E:T ratio of 100:1. However, Ad-LacZ and non-treated DCs induced CTLs could not lyse U251 cells (Fig. 2).

### None specific lysis assay

**Fig. 3:**
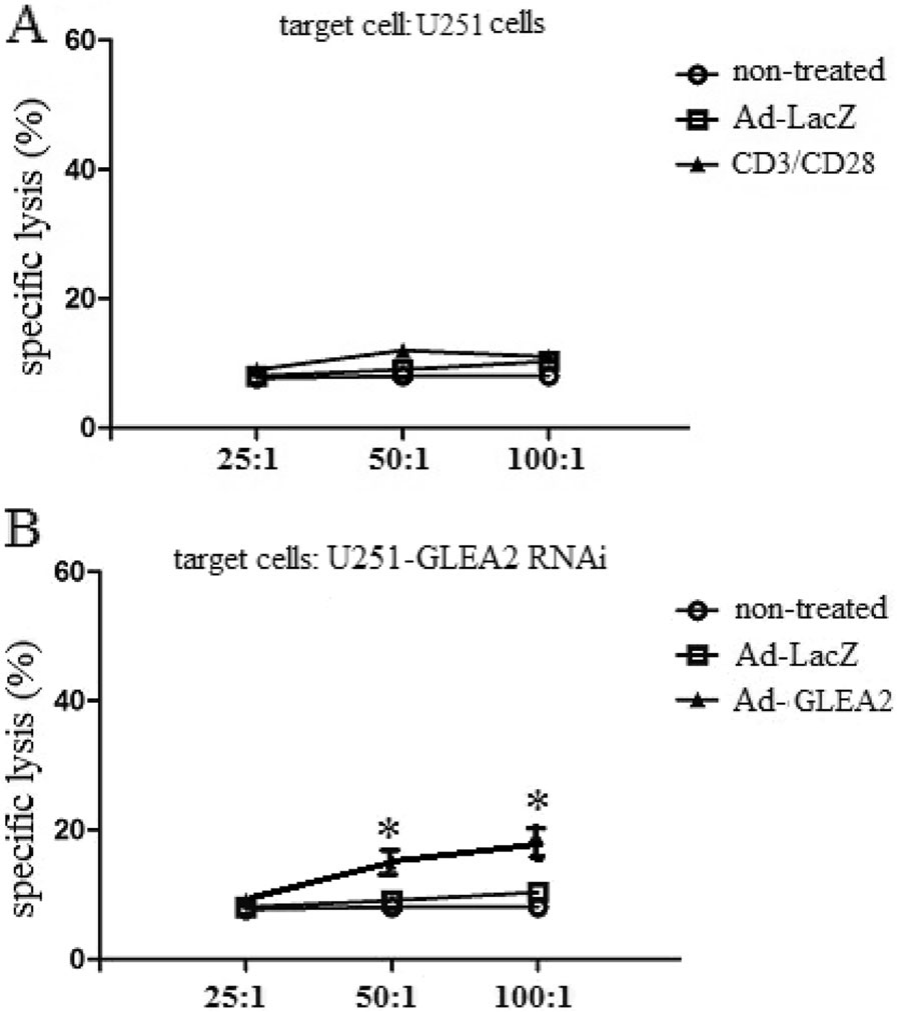
GLEA2-specific CTLs were tested for their ability to lyse U251 cells expressing GLEA2. **a** GLEA2-specific cytotoxic T lymphocytes (CTLs) were generated in vitro with 5 μg/ml of anti-CD3 antibody (clone 17A2; Biolegend) pre-bound to the culture plate and 2 μg/ml of soluble anti-CD28 antibody (clone 37.51; Biolegend) at 37 °C for 3ds. GLEA2-specific CTLs were tested against U251 cells. **b** GLEA2-specific cytotoxic T lymphocytes (CTLs) were generated by weekly stimulation of nonadherent peripheral blood lymphocytes with irradiated, autologous DCs transduced with Ad-GLEA2. GLEA2-specific CTLs were tested against U251 cells lacking GLEA2 expression. CTLs generated from Ad-LacZ transduced DCs and effector cells generated from T lymphocytes stimulated by PBS were used as controls. Experiments performed in triplicate showed consistent results.

To explore whether adenovirus transduced DCs could elicit GLEA2 specific CTLs, we utilized CD3/CD28 activated DCs to elicit CTLs. We found that CTLs elicited by CD3/CD28 activated DCs could not lyse U251 cells (Fig. 3a). In addition, to explore whether GLEA2 specific CTLs would lyse nonspecific target cells, U251 cells lacking GLEA2 expression were utilized as a target cells. We found that lysis ratio of GLEA2 specific CTLs to U251 cells lacking GLEA2 expression was significantly attenuated in vitro (Fig. 3b). The data suggested that GLEA2 specific CTLs had no immune system side effects on none specific target cells.

### Induction of TNF-α-producing CTLs

**Fig. 4:**
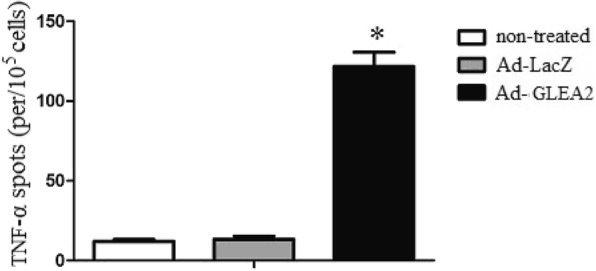
Specific TNF-α by ELISPOT assay GLEA2-specific cytotoxic T lymphocytes (CTLs) were generated in vitro by weekly stimulation of splenocytes with irradiated autologous DCs transduced with Ad-GLEA2. The single-cell suspensions of splenocytes were prepared as effector cells to quantify specific TNF-α releasing cells. Triplicate experiments showed consistent results. Compared with controls,*P < 0.05.

To explore whether adenovirus transduced DCs could induce CTLs to generate cytokines, we analyzed the TNF-α by ELISPOT assay. GLEA2-specific cytotoxic T lymphocytes (CTLs) were generated in vitro by weekly stimulation of peripheral blood lymphocytes with irradiated autologous DCs transduced with Ad-GLEA2. The single-cell suspensions of peripheral blood lymphocytes were prepared as effector cells to quantify specific TNF-α releasing cells. The results demonstrated that Ad-GLEA2 transduced DCs could induce high frequencies of TNF-α-producing T cells compared with control groups (Fig. 4). The data suggested that Ad-GLEA2 transduced DCs promoted CTL activity.

### Inhibition of tumor growth

**Fig. 5:**
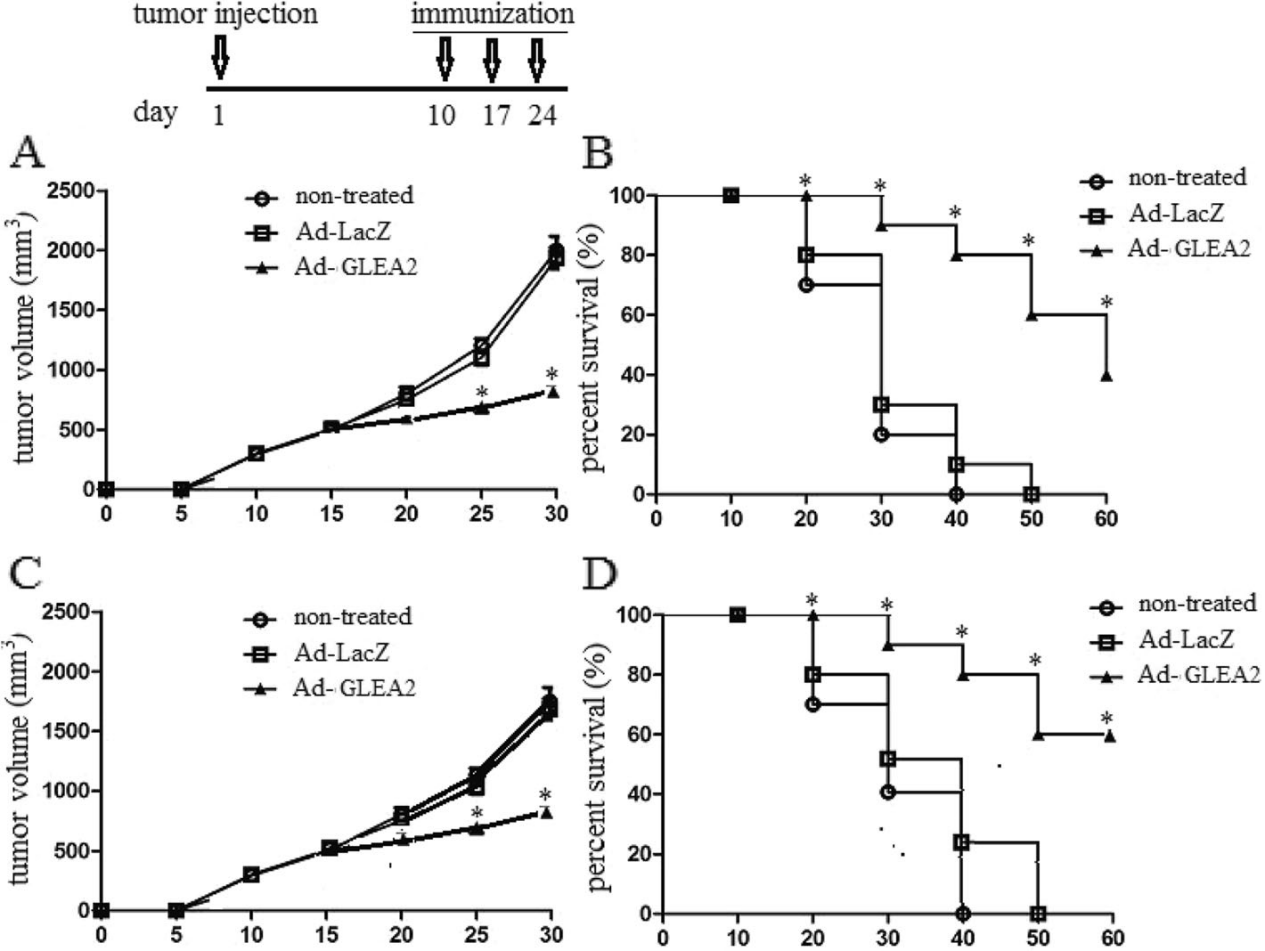
Primary tumor growth and the average lifespan of mice. The mice (*n* = 10) were challenged with an s.c. injection of 1 × 10^6^ U251 cells into the left flank to induce a primary tumor model. **a** and **b**. 10 days after tumor challenge, Trimera mice were immunized s.c. in the base of the tail with 1 × 10^6^ transduced DCs in 100 μl PBS for three times once a week. The average tumor volume and average lifespan of mice are depicted. **c** and **d**. 10 days after tumor challenge, Trimera mice were injected i.v. of 1× 10^7^ lymphocytes. Control mice received the same volume of PBS. The tumor volume and mean lifespan of mice were observed. Experiments performed in triplicate showed consistent results

To identify whether the Ad-GLEA2 could exert anti-tumor capability in vivo, the mice (*n* = 10) were challenged with an s.c. injection of 1 × 10^6^ U251 cells into the left flank to induce a primary tumor model. 10 days after tumor challenge, Trimera mice were immunized s.c. in the base of the tail with 1 × 10^6^ transduced DCs in 100 μl PBS for three times once a week. Tumor volume and lifespan of the mice were observed. We found the tumor volume expanded rapidly after 25 days of tumor challenge in the PBS and Ad-LacZ groups. However, in the Ad-GLEA2 group, the tumor volume expanded slowly (Fig. 5a). In addition, the mean lifespan of tumor bearing mice in the Ad-GLEA2 group was prolonged significantly (Fig. 5b). In addition, to further analyze whether elicited T cells has the potential to anti-tumor, we transferred T cells into tumor bearing mice, and detected the tumor volume and mean lifespan. The data demonstrated that Ad-GLEA2 elicited T cells had ideal anti-tumor efficiency (Fig. 5c and d). These data suggested that Ad-GLEA2 trasduced DCs had the anti-tumor capability in vivo.

## Discussion

Glioma is one of the most common malignancies in the world. Resection, chemo-embolization, surgery ablation and radiotherapy are the most commonly curative treatments in the clinic [22–24]. However, the postoperative 2-year recurrence rate remains high and the long-term survival prognosis of patients is still poor [25–27].

Immunotherapy has been regarded as a promising therapeutic strategy for malignant tumors in the past years, which has the advantages of high anti-tumor efficiency and weak side effects [28–30]. One of the opinions for tumor immunotherapy is tumor-associated antigen (TAA) vaccine that could induce specific and long-lasting immune response [30–32]. GLEA2 is a novel antigen which is expressed in glioma and induces an immune response in more than 43% of all glioma patients [33]. However, the immunotherapy strategy based on GLEA2 has not been reported.

Firstly, DCs were transduced with Ad-GLEA2, and GLEA2 expression was detected. The results suggested that Ad-GLEA2 could induce into DCs and mediate GLEA2 protein expression. Secondly, Ad-GLEA2 transduced DCs were utilized to elicit GLEA2-specific CTL activity in vitro. The data suggested that Ad-GLEA2 transduced DCs promoted CTL activity without obvious immune system side effects. Lastly, the tumor bearing mice were immunized with Ad-GLEA2 transduced DCs, and the tumor volume and lifespan of the mice were observed. These data suggested that Ad-GLEA2 transduced DCs had the anti-tumor capability in vivo.

## Conclusions

Taken together, our data suggested that DCs transduced with Ad-GLEA2 could elicit a robust anti-tumor immune response against glioma in vitro and in vivo.

## Supplementary information

**Additional file 1.** **Figure S1.** The represent FACS figures of DCs expressed high level of CD11c and MHC Class II.

## Data Availability

Please contact author for data requests.
