# Supplementary figures and images for: Dendritic cells transduced with glioma-expressed antigen 2 recombinant adenovirus induces specific cytotoxic lymphocyte response and anti-tumor effect in mice
Source: J Inflamm (Lond). 2020 Jan 31;17:3. doi: 10.1186/s12950-020-0239-6 (PMC6995099; doi:10.1186/s12950-020-0239-6)

Additional file: Figure S1


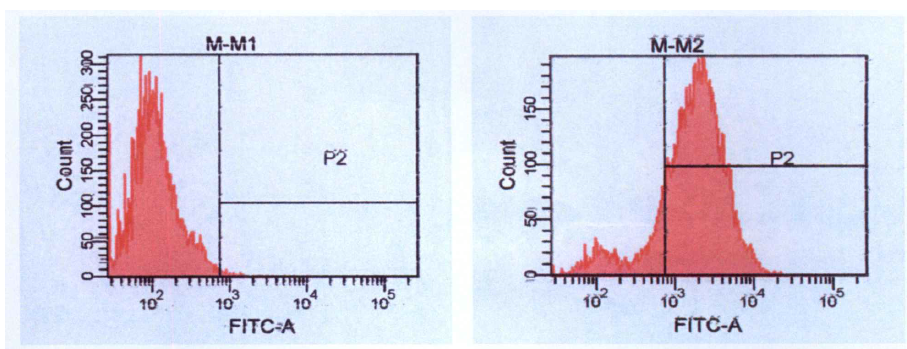


ISOPYPE CD11c


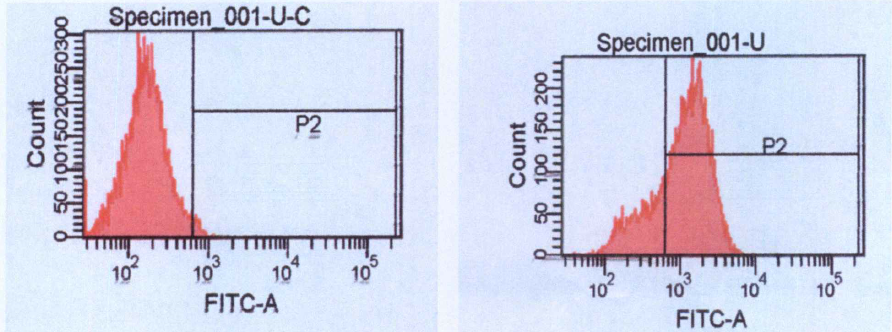


ISOTYPE MHCII.

Supplement: Supplementary file 1 — Additional file 1. Figure S1. The represent FACS figures of DCs expressed high level of CD11c and MHC Class II. [file 12950_2020_239_MOESM1_ESM.doc]
